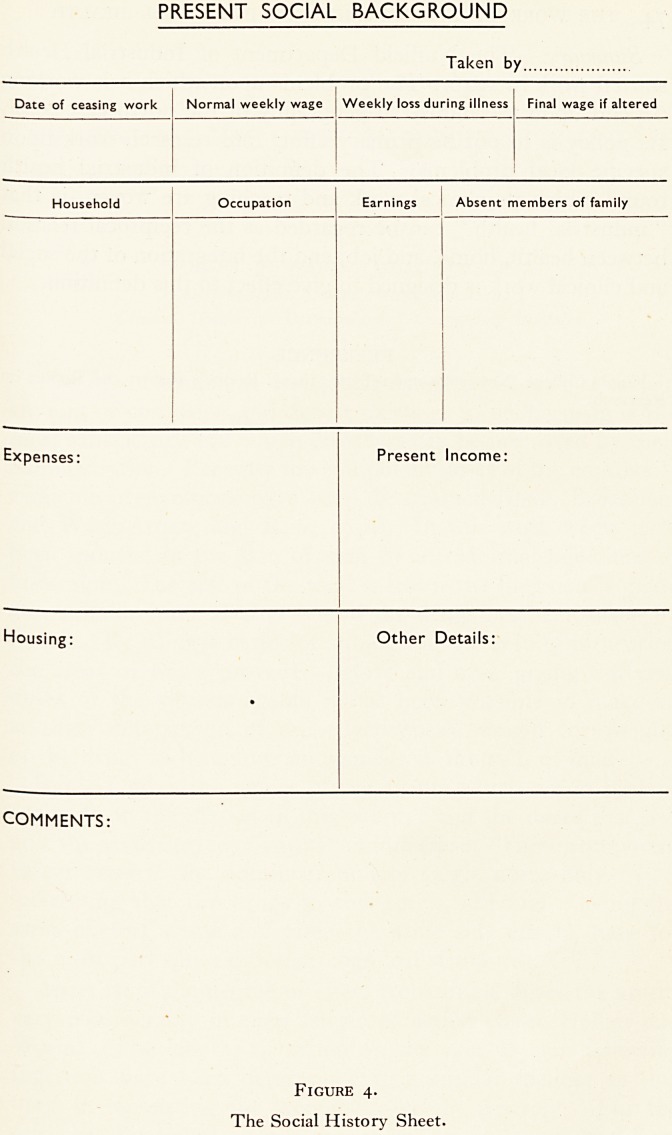# The Work of a Department of Industrial Health
*Based upon an address to the Bristol Medico-Chirurgical Society on January 11th, 1950, and a lecture delivered before the Divisions of Medicine and Laboratories, Radcliffe Infirmary, Oxford, February 15th, 1950.


**Published:** 1950-07

**Authors:** R. C. Browne

**Affiliations:** Nuffield Professor of Industrial Health, University of Durham, King's College, Newcastle-upon-Tyne


### The Bristol Medico-Chirurgical Journal

*A Journal of the Medical Sciences for the*

### West of England and South Wales

*" Scire est nescire, nisi id me Scire alius sciret."*

JULY, 1950

### *THE WORK OF A DEPARTMENT OF INDUSTRIAL HEALTH

BY

R. C. BROWNE, D.M., M.R.C.P.

*Nuffield Professor of Industrial Health,*
*University of Durham, King's College, Newcastle-upon-Tyne*

The first step towards the foundation of a Department of Industrial Health was made in 1944 by an inquiry from the Nuffield Foundation to the King's College division of the University of Durham, asking what future plans for forming such a department existed, and whether the university would accept financial help to bring these to fruition. Work started in June, 1946, when the department consisted of a room containing a few packing-cases. Now, it is clearly important that heavily industrialized areas should have first priority for the foundation of such departments, but the work they ultimately have to do depends very largely upon the " macroscopic " industrial geography of the surrounding area, and the ease with which they

* Based upon an address to the Bristol Medico-Chirurgical Society on January 11th, 1950, and a lecture delivered before the Divisions of Medicine and Laboratories, Radcliffe Infirmary, Oxford, February 15th, 1950.

can do it relates closely to the "microscopic" academic geography, because frequent consultation with other scientific departments is of great importance. Figure 1 shows the salient points of the industrial geography of the north-east coast, and marks clearly the importance of coal-mining and shipbuilding and, by implication, all the industries that go with them. For example, ships need painting, so there are paint works; boilers need lagging with a heat-resisting material, so there are factories making asbestos lagging, and so on.

### DEPARTMENTAL POLICY

The policy of the department is bound up to a considerable extent with its definition of "industrial health", which is defined shortly as the reciprocal relation between health, home, and job. It is better to use an over-simplified definition which can act as the centre around which the rest of the work crystallizes, than an elaborate generalization to cover almost everything. It is the policy to take a synoptic or holistic view of the work, combining on the one hand clinical with social medicine, and on the other the laboratory approach, and to select either the experimental or the observational method, depending upon the nature of the problem. The terms of reference under which the department was founded suggest that it should give priority in its efforts to work upon specific problems. This is usually called "research", but, as is pointed out in the Rector's (1949) recent report, this is rather a pretentious word, if only because real research usually consists of about 2 per cent. inspiration and 98 per cent. perspiration. The policy is to concentrate on the local problems in industries which have the greatest environmental difficulties, and which employ the greatest numbers. Next come teaching and a certain amount of routine medical work. Both compensation and medico-legal work are avoided, because it is most important for a university department to maintain an independent outlook, and it cannot do this by taking sides in such disputes. An attempt is made therefore to maintain a fair and independent attitude between employer and employed, and, as an example of this practice, it will be seen later how some pieces of work are organized through the help of the employers and others through the help of the trades unions.

The industry which employs the greatest numbers and which has the greatest environmental difficulties in the area is coal-mining, and here work is being done upon some facets of the problem of coal-miners' nystagmus. We are attempting to answer the question, " how do men with this disease differ from normal miners?", by studying their social and occupational histories, and by relating these to comparable histories of normal men. An attempt is also being made to answer the questions, " can oscillations of the eyes be produced in animals by exposing them to prolonged darkness? ; and if so, can they be cured by exposure to light, and how much light, and for how long? " This problem of nystagmus is being studied in collaboration with the Departments of Psychological Medicine and of Physiology, and with the Sunderland Eye Infirmary. So far only a partial retrospective study of old cases of nystagmus has been made. The suggestion from this is that, taking them by and large, patients with this disease are very little different from ordinary men; that the prognosis for return to underground work and for remaining at it after five years is poor (only 4 per cent. of them manage this); and that the other 96 per cent. need rapid resettlement to standing-up work in good light. The second coal-mining problem which is being studied is coal-miners' pneumoconiosis, and here of course it is most important to relate our work to that of the Medical Research Council's unit in South Wales, which is putting so much effort into investigating this disease. The prevalence of the disease in the North-East is unknown. It has been said that County Durham was particularly free from it; but in the last three years this department has seen nearly 300 clinical cases of the condition, and it is now important to carry out surveys to get an idea of its real prevalence in some of the collieries. The social consequences of the disease in this area of England are being investigated, and how they relate to the social consequences of bronchitis and emphysema which are equally common in coal-miners. Finally, an attempt is being made to work out the principles on which patients with pneumoconiosis should be advised. It is one thing to see this problem in an atmosphere of what might be called " pure research ", but it is quite another to have patients before one in an out-patients' clinic, asking for advice which must be related not only to the natural history of the disease, but to its social and occupational consequences as well.

Another very important industry in this area is that of shipbuilding, whether viewed from the clinical or the social angle. Statistical surveys are therefore being made in two shipyards on the Tyne, in which branch offices staffed by members of the department are established. In other words, they are in the environment which they are studying, and are attempting to relate, statistically, the incidence of accidents and absences to job. The outstanding thing that strikes a visitor to a shipyard on going into the sick quarters and looking at the sick book, is the very high incidence of trauma—actual damage to the body—in relation to the number of workpeople. Nearly all shipbuilding is done out of doors, and a large part of the work has a big manual element in it.

Now to turn to a laboratory problem of the back-room type. There is in industry a certain relation between a man and the machine which he is controlling, and this relation may break down in a number of different ways. He may have an accident, the output may fall off, or the amount of spoilt work which he turns out may go up, and so on. There is a number of situations in which men are controlling machines in response to the display of some sort of information, which tells the controller what the machine is doing. An example of this is the ship's engineer, who controls his engine in response to the engine-room gauges. The winding engine-man in a colliery is another example, and another, the air pilot who is flying in cloud or at night, and who cannot see the horizon. These three machine controllers have to be told what is happening by some type of indicator, and the problem in which we are interested is: how should such indicators be designed so as to produce the greatest speed and accuracy of response on the part of the machine controller? A small piece of this larger problem is being worked upon, and it is this : given a numerical indicator, should it be arranged as a vertical or horizontal strip, or as a circular dial? In order to study this kind of question, it is necessary to make an apparatus which produces movements of a pointer over the dial, in response to which the human subject of the experiment has to move a control knob or handle; that type of display which produces the

FIGURE I.

Diagram of the industrial geography of the North-East Coast. The main towns are hatched and the coal mining area is within the the triangle. The population in thousands for the three most important industries and in the three main river valleys is shown.

FIGURE 2.

Industrial erosion of the teeth.

greatest speed and accuracy of response is the best. The apparatus for this kind of work consists essentially of three parts, a machine for producing an unpredictable movement of the pointer, a control and display panel, and an ink writer for graphing what the pointer does and how the subject responds to it.

A further problem of a more narrowly industrial type which is being studied is the erosion of human teeth by certain dusts (Figure 2). This work started through the clinical observation that girls working in a certain room in a local factory showed a peculiar erosion of the enamel, and at a later stage, the dentine of their teeth. A visit to the factory made it obvious that there was a continuous dust-cloud in this workroom, which was not present in other workrooms in the factory; and a survey showed that this peculiar dental disease was confined, in fact, to this room. A dust-sampling survey was carried out in all parts of the room, and a quantitative and qualitative analysis of the dust was made. The observed clinical disease can now be linked with what is in the dust. And it now remains to complete the logical circle by producing the disease *in vitro*, which will also help to answer the question, " what is the greatest concentration of the chemical in the air which can be allowed before erosion takes place? " This piece of work illustrates the complete cycle of steps in research in industrial medicine. First, clinical observation; secondly, the correlation with the observed environment; thirdly, putting the teeth artificially in the observed environment and seeing if the same thing happens; and fourthly, the production of a time-concentration curve for the action of the noxious body upon the teeth.

### TEACHING

In teaching the departmental policy is to give priority to the undergraduate medical student, because it is felt that every doctor should understand something about the reciprocal relation between health, home and job, and that this is by no means merely a postgraduate subject. Four methods are used in teaching: the tutorial, the field visit, the out-patient department and the lecture, in roughly that order of effectiveness. Undergraduate tutorials are given twice a week throughout the year to those students who are doing their senior medical clerkships, in a side room of one of the wards of the department of medicine, and the teaching is firmly based upon a clinical platform. The students are taught to take three types of history from the patient, the clinical history, the occupational history and the social history; to make three diagnoses, clinical, occupational and social; and then to plan the management (a wider conception than " treatment ") of the patient by fitting together the information from these three sources. Only small numbers—six or eight students—are taught at a time, and they are always taught on a real clinical problem for which the department of industrial health has clinical responsibility, and a social worker is always present to present the social side of the problem. This type of teaching, although integrative and concentrated, is limited by the fact that the problems being discussed have been, to a certain extent, prepared beforehand by the teacher, or at any rate considered by him. Clinical students are therefore taught in the out-patient clinic of the department, where they see their seniors practising what they preach in the wards. Here the clinical and occupational histories are taken by the physician, the clinical examination is then made, and the social history is then taken by the social worker, who comes in and presents it before the students, so that they see the whole problem. The patient returns next week after the necessary investigations have been made, and the students see how the advice given is based upon all the information previously obtained. A course of lectures is given to the final-year students on the relationship of the environment to health, which attempts to bring together the various loose ends of the subject; and a share is taken in teaching the postgraduates taking their diploma in public health, which, in the University of Durham, contains an element of industrial health, the department of which also offers a course in statistics to these postgraduates. There is one other type of teaching which is given, and that is to the third-year engineering students, upon the relationship of engineering to health, because many of these students will be designing machinery, which either has to be controlled by human beings or which affects them in one way or another, and in later life they will become factory administrators with, in fact, more control over the health of their workpeople than the doctor, who only tends to be called in when things go wrong.

As well as the specific problems and the teaching, there is an element of what might be called routine work, which acts as a " cement ", sticking the activities of the department together. Twice a week in-patient rounds are done together with the house physician and the social assistant. It is often objected that an almoner has no place at the bedside, but when a decision has to be made about the management of a particular patient, including of course the social and occupational management, this decision can only be made well when all the information, clinical, social and occupational, can be delivered at one place and at one time, at what is really a small case-conference. This is not, of course, done over the patient's bed or even within earshot of the patient, but it is done on the spot, so that all the people interested in the patient's welfare know what the others are thinking. Two out-patient clinics are conducted, and for these a new type of note-keeping system has been developed, which gives more prominence to the social and occupational facets of the patient than do the notes usually used (Figures 3 and 4). A large proportion of the work of these two clinics is follow-up work, because in them are seen cases of coal-miners' pneumoconiosis, and these men need to have their chests X-rayed at least annually, together with an assessment of their clinical and occupational state.

### STAFF

The staff needed to carry this load of work consists of a first assistant, who is a clinician with a special interest in chest disease, matching the main clinical problem of the department; a social assistant, who is an almoner; a statistician (with two statistical clerks) whose primary objective is the statistical survey of shipyard workers, but who also gives statistical advice to anyone in the medical faculty who needs it; a physiologist, who is carrying out research on indicator display with the assistance of a Medical Research Council grant; a chemist, working on the tooth-erosion problem, who is assisted by a Luccock Research Fellowship; a social surveyor, in charge of the social survey of a shipyard town; and a secretarial staff of two, one of whom primarily deals with the clinical secretarial work, and the other with the social secretarial work together with the organization of the out-patient clinic.

### HISTORY (OCCUPATIONAL)

Name and nature of present job:

Past employment or unemployment

| Age | Job | Age | Job |
|---|---|---|---|
| | | | |

FIGURE 3.

The Occupational History Sheet.

### PRESENT SOCIAL BACKGROUND

Taken by....................

| Date of ceasing work | Normal weekly wage | Weekly loss during illness | Final wage if altered |
| --- | --- | --- | --- |
| | | | |

| Household | Occupation | Earnings | Absent members of family |
| --- | --- | --- | --- |
| | | | |

| Expenses: | Present Income: |
| --- | --- |
| | |

| Housing: | Other Details: |
| --- | --- |
| | |

COMMENTS:

FIGURE 4.

The Social History Sheet.

*Summary*.—The Nuffield Department of Industrial Health started work in 1946. The problems upon which it is working depend upon the industrial geography of the north-east coast. Its policy is to put its primary effort into research work upon specific local problems. The definition of industrial health round which its clinical work and teaching are woven, is that "industrial health" can be regarded as the reciprocal relation between health, home, and job, and the integration of the social and clinical work is designed to give effect to this definition.

### REFERENCE

King's College, Newcastle-upon-Tyne (1949): Rector's Report and Report on Research.

## Figures and Tables

**Figure 1. f1:**
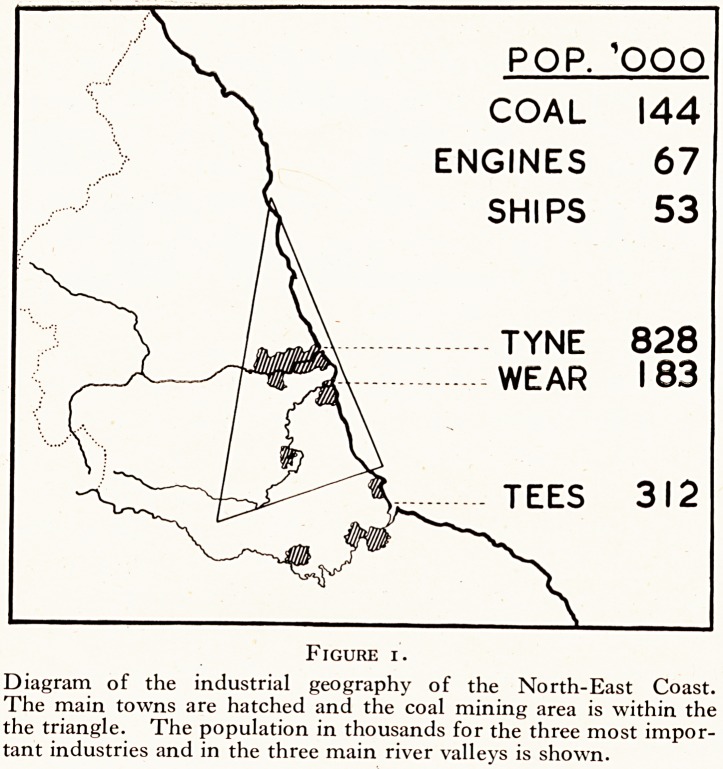


**Figure 2. f2:**
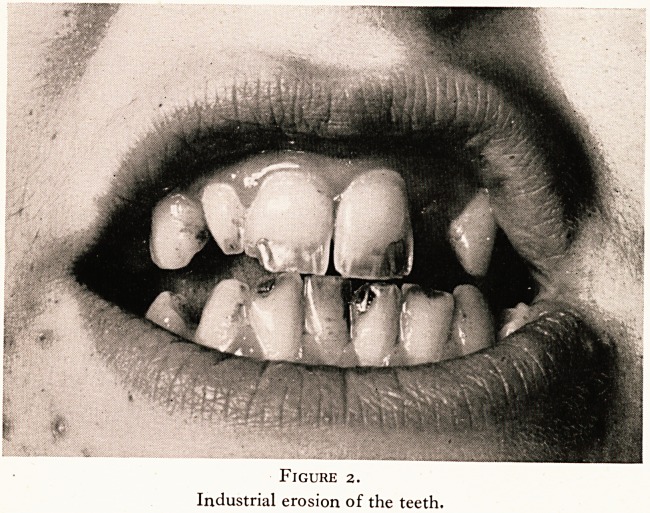


**Figure 3. f3:**
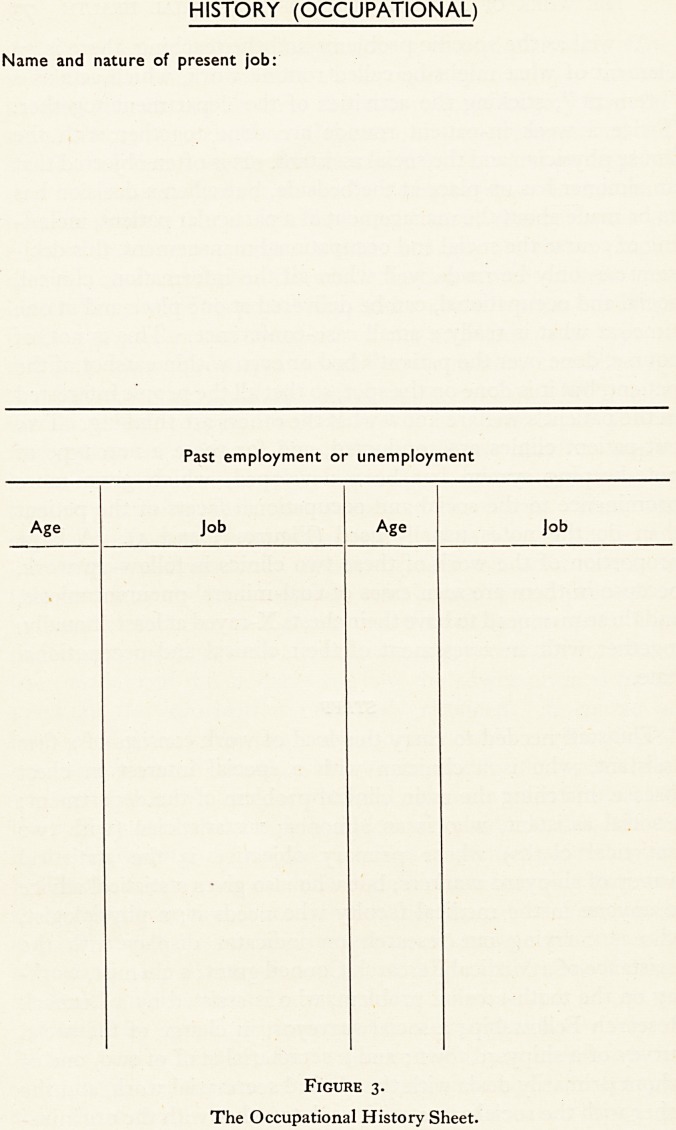


**Figure 4. f4:**